# The international food unit: a new measurement aid that can improve portion size estimation

**DOI:** 10.1186/s12966-017-0583-y

**Published:** 2017-09-12

**Authors:** T. Bucher, M. Weltert, M.E. Rollo, S.P. Smith, W. Jia, C.E. Collins, M. Sun

**Affiliations:** 1 Department of Health Sciences and Technology, ETH Zurich, Universitätsrasse 16, 8092 Zurich, Switzerland; 2 Priority Research Centre for Physical Activity and Nutrition, Faculty of Health and medicine, The University of Newcastle, Newcastle, Australia; 3 School of Electrical Engineering and Computing, The University of Newcastle, Newcastle, Australia; 4 Departments of Neurosurgery, University of Pittsburgh, Pittsburgh, USA

**Keywords:** Portion size measurement aid, PSMA, PSEM, Volume and capacity training, Standardisation, Dietary assessment, Food shape, Automated food volume recognition, Food intake reporting

## Abstract

**Background:**

Portion size education tools, aids and interventions can be effective in helping prevent weight gain. However consumers have difficulties in estimating food portion sizes and are confused by inconsistencies in measurement units and terminologies currently used. Visual cues are an important mediator of portion size estimation, but standardized measurement units are required.

In the current study, we present a new food volume estimation tool and test the ability of young adults to accurately quantify food volumes. The International Food Unit™ (IFU™) is a 4x4x4 cm cube (64cm^3^), subdivided into eight 2 cm sub-cubes for estimating smaller food volumes. Compared with currently used measures such as cups and spoons, the IFU™ standardizes estimation of food volumes with metric measures. The IFU™ design is based on binary dimensional increments and the cubic shape facilitates portion size education and training, memory and recall, and computer processing which is binary in nature.

**Methods:**

The performance of the IFU™ was tested in a randomized between-subject experiment (*n* = 128 adults, 66 men) that estimated volumes of 17 foods using four methods; the IFU™ cube, a deformable modelling clay cube, a household measuring cup or no aid (weight estimation). Estimation errors were compared between groups using Kruskall-Wallis tests and post-hoc comparisons.

**Results:**

Estimation errors differed significantly between groups (H(3) = 28.48, *p* < .001). The volume estimations were most accurate in the group using the IFU™ cube (Mdn = 18.9%, IQR = 50.2) and least accurate using the measuring cup (Mdn = 87.7%, IQR = 56.1). The modelling clay cube led to a median error of 44.8% (IQR = 41.9). Compared with the measuring cup, the estimation errors using the IFU™ were significantly smaller for 12 food portions and similar for 5 food portions. Weight estimation was associated with a median error of 23.5% (IQR = 79.8).

**Conclusions:**

The IFU™ improves volume estimation accuracy compared to other methods. The cubic shape was perceived as favourable, with subdivision and multiplication facilitating volume estimation. Further studies should investigate whether the IFU™ can facilitate portion size training and whether portion size education using the IFU™ is effective and sustainable without the aid. A 3-dimensional IFU™ could serve as a reference object for estimating food volume.

**Electronic supplementary material:**

The online version of this article (10.1186/s12966-017-0583-y) contains supplementary material, which is available to authorized users.

## Background

Larger portion sizes increase energy intake in children and adults [1–3], a phenomenon termed the portion size effect [4]. Choosing appropriate portion sizes and being aware of amounts consumed is a critical skill to assist with weight control, improve health and lower chronic disease risk [5]. Not being able to accurately estimate food portions makes it problematic for implementing dietary recommendations. In addition, health care professionals cannot generate an accurate assessment of a patient’s food and nutrient intake [5].

People generally have difficulties assessing food portion sizes [6–8] and consumers are confused as measurement units and terminologies are used inconsistently and differ internationally [9].

Dietary recommendations are often communicated in cup measures. For example, in Australia a standard serve of cooked vegetable is half a cup, with five standard servings recommended daily [10]. However, cup measures differ internationally. One cup in Japan is 200 ml, a traditional Japanese cup is 180 ml (the gō), a Canadian cup is 227.3 ml (8 imperial oz), a U.S. Customary cup is 236.6 ml (8 oz), a US legal cup is 240 ml, the Imperial cup (UK) is 285 ml and the metric cup (Commonwealth countries) is 250 ml. Conversely, many European countries such as Switzerland, Germany or the Netherlands do not use cups, but refer to grams and milliliters (for liquids) in recipes, on food labels and within dietary recommendations.

Spence et al. found consumers mentioned that household measurements were open to interpretation, with poor recognition of actual serving quantities specified in metric (e.g. grams) or imperial (e.g. ounces) measurements [9]. In their study, participants recalled inconsistent serving size information for specific foods and food groups provided by the public, private and voluntary sectors (e.g. dietitians, weight loss communities, food labels) [9]. Variation in terminologies, measurement units and recommendations cause confusion and lack of clarity on recommended serving sizes was the major barrier to appropriate food portion size selection in adults [9, 11].

**Fig. 1:**
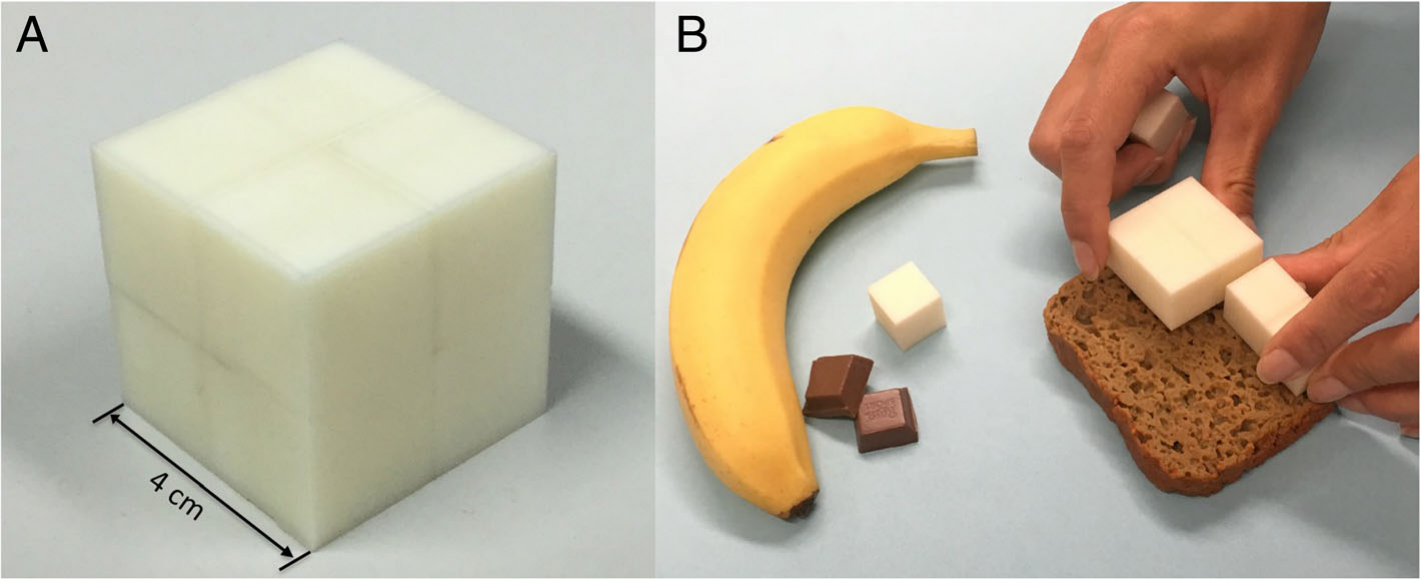
**a** The International Food Unit™ (IFU™): A 4x4x4 cm cube with a volume of 64cm3 (64 ml), which can be subdivided into 8 smaller cubes (2x2x2cm = 8 cm3 (8 ml)) to estimate smaller volumes **b**. The present study tested the performance of the IFU™ as a measurement aid

To assist with countering the obesity epidemic, portion size interventions and education are acknowledged strategies to improve dietary habits and reduce overall energy intake at the population level [12]. A systematic review of the impact of portion education and training interventions on dietary intake concluded that interventions can improve adults’ ability to estimate portion sizes [13]. Visual cues are an important mediator of portion size estimation and education [4, 14]. However, standardized international terminologies, food measures and aids are needed to avoid confusion and facilitate education strategies related to dietary recommendations [11]. Rather than ‘cup’ measures, new consumer-focussed methods that standardize food volume estimation using SI units (e.g. cubic metres = m^3^) or the Centimetre-Gramm-Second (CGS) system (e.g. cubic centimetres = cm^3^) should be considered [11]. Standardised aids for food volume measurement based on SI units may facilitate international food portion measurement and surveillance and portion control strategies [11]. There is an urgent need to develop a standardised food volume unit, which is accepted across cultures with unique intakes [11]. Here we propose a 4x4x4 cm cube with a volume of 64 cm^3^ (64 ml) that can be subdivided into eight 2 cm sub-cubes for estimating smaller portions, as a potential International Food Unit IFU™ and food volume measurement aid (Fig. 1).

The IFU™ was developed by an international team of dietitians, nutrition scientists, bioengineers and computer scientists. The dyadic division scheme of the cube size follows the use of the binary system in computer systems since binary dimensional increments facilitate portion size education and training, memory and recall, and computer processing, which is binary in nature. The cubic shape is important for understanding of the mathematical volume concept by allowing people to visually correlate a cube of defined volume with an irregularly shaped object (here a food object). Another important feature of the IFU™ as a reference object is that it is smaller than a standard measuring cup and more similar to actual consumption units, such as a portion of meat recommended for usual consumption. The IFU™ can easily be converted into volumes of existing household measurement cups by multiplication (e.g. 4 IFUs™ are similar to the US and the metric cup, and 3 IFUs™ are similar to the Japanese cup). In addition, subdivision and multiplication of the cube have a straightforward visual correspondence hence the IFU™ facilitates both volume estimation and observation from the screen of a computer or a mobile device. A cube, which can be subdivided into smaller cubes, further allows easier measurement of foods with different shapes and volumes since it is already in volumetric units. This could be useful for food volume and portion size education and training. Moreover, the cubic shape is strategic, as it can serve as a 3D reference object on food photographs or within smartphone applications.

The aim of the current study was to test the performance of the IFU™ for food volume estimation.

## Methods

### Study sample

Written informed consent was obtained from all subjects prior to participation. Participants were recruited between June and September 2016 via flyers, handouts and social media in Newcastle, NSW, Australia. People were included if they were aged ≥18 years, did not have a university degree major in nutrition or dietetics and did not have food allergies or intolerances.

### Experimental procedure

Participants were invited to estimate 23 specific food portions, 17 different foods, three with three differing portions sizes (Additional file 1: Figure S1 and Additional file 1: Table S1). A team of nutritionists and dietitians selected foods commonly consumed in Australia. A range of food shapes and sizes were included as these were considered to potentially influence estimation errors. To minimise food preparation and food waste, authentic and validated food replicas [15] previously used for portion size and serving studies [16–19] were used (Döring GmbH, Germany). The exception was for nuts and dried fruit mix, which was purchased at a local retailer (Woolworths Limited, Australia), as no good quality replicas existed. Foods were classified into Australian Guide to Healthy Eating (AGHE) food groups (National Health and Medical Research Council (NHMRC), 2016). For rice, chicken and French fries, three different portion sizes to test the impact on estimation accuracy [20–22] were included. These three foods were selected as they do not come in predefined portions but have different shapes. Portion sizes for continuous (uncountable) foods were informed by a previous study on adult perceptions of small, medium and large portion sizes [23], one piece or slice was used for fruit and bread. For French fries, the small, medium and large portions served in McDonald’s were used [24].

Subjects were manually randomised to one of four experimental conditions, which differed in the portion size estimation aid provided (Additional file 1 **:** Table S2). Therefore, a number was drawn from a box containing cards with numbers for the four conditions. Two different boxes were used for males and females to balance gender between conditions. Group 1 used one IFU™ (64 mL) to estimate food volumes. Group 2 used a modelling clay cube of the same volume. Group 3 used an Australian cup (250 mL), while group 4 estimated food weights with no aid. Weight instead of volume estimation was chosen in group 4 as estimation aids generally improve portion size estimation and we would have expected large errors in volume estimation without a reference object. However, consumers might be more familiar with food weight given this is usually presented in the information on food labels and from grocery shopping for foods sold by weight.

Food portions were presented on the same size plates (IKEA Australia, Australia). Milk was presented in a glass on the plate. Participants received one plate at a time to assess, with no direct comparison between items allowed. Foods were presented in random order to control for fatigue and learning effects [21, 25]. Subjects were not allowed to touch the foods, but were allowed to handle the estimation aids, e.g. they could subdivide the IFU™ into smaller cubes or reshape the modelling clay cube.

Volume estimates had to be reported relative to the estimation aid and participants were free to report estimates in decimals, fractions or percentages. For data analysis, all values were converted into decimals. In the condition with no aid, estimates were reported in grams or ounces based on individual choice.

#### Survey

After participants estimated all food portions, they were asked to complete a questionnaire (Qualtrics, LLC, Utah, USA) assessing potential confounding variables (numeracy, cooking frequency, use of measuring aids at home, knowledge of dietary guidelines (AGHE), consumption frequency of experimental foods and hunger), which could influence the accuracy of portion size estimates. Participants’ subjective numeracy was assessed using the Subjective Numeracy Scale (SNS) [26]. They were asked how often they cook from scratch, bake, or consume ready-made meals. Further, they were asked how often they use measuring cups, scales or other estimation aids. These questions were answered using a five-point scale (1 = Daily; 2 = Several times per week; 3 = Several times per month; 4 = Once a month or less; 5 = Never).

Knowledge of dietary guidelines was assessed with one question ‘Are you familiar with the Australian Guide to Healthy Eating (AGHE) standard serve sizes? (No; I have heard about them; Yes, I know them). Consumption frequency of each experimental food (*n* = 17) was measured on a six-point scale (1 = ≥5 per week; 2 = 2-4 per week; 3 = Once per week; 4 = 1-3 per month; 5 = < 1 per month; 6 = Never). Hunger level was measured on a six-point scale (1 = Not hungry at all; 6 = Very hungry). Data on sex, age, self-reported weight and height, education and country of birth was collected. Usability of the estimation aids was evaluated by asking whether the aid helped them to estimate portion sizes and if it was easy to use (1 = strongly disagree; 5 = strongly agree), which food they found the easiest to estimate and which the most difficult. The reasons for their choice were assessed with an open question.

### Statistical analysis and measures

#### Estimation errors

Estimates with the IFU™^,^ the modelling clay and the measuring cup were compared to the actual food volume relative to the aid and weight estimates made without an aid to the weight of the corresponding real food in grams. The relative estimation error was calculated as follows: ([estimated amount or volume– actual amount or volume]/actual amount or volume) * 100. Relative estimation errors were calculated for each food as well as a group mean across all 17 foods in order to assess estimation accuracy across the four experimental conditions. For the foods presented in three different portion sizes, the relative estimation error of the medium portion was used to calculate the mean relative error. Here, the term estimation error refers to the relative estimation error, unless stated otherwise. For the purpose of comparison with specific studies in literature, absolute errors were calculated (| (estimated amount or volume– actual amount or volume) |/actual amount or volume) * 100) and classified into within 25% and within 75% range.

Statistical analysis was performed using IBM SPSS Statistics Version 23 (SPSS Inc., Chicago, IL, USA). The Kolmogorov-Smirnov test was used to test whether data was normally distributed; the Levene’s test to test for homogeneity of variance. Normally distributed data was summarised as mean (*M*) and standard deviation (*SD*); non-normal data as median (*Mdn*) and interquartile range (*IQR*). The significance level was set at *P* < .05. Distribution of continuous variables was compared between the four experimental conditions using the Kruskal-Wallis test *(H)* and categorical variable distribution using Pearson’s chi-square (χ^2^) test. Differences between portion sizes were investigated using Friedman’s ANOVA, as each individual estimated all portions and the data is dependent. Post-hoc comparisons were performed for independent conditions using the Mann-Whitney test and for related groups using the Wilcoxon signed-rank test. The Bonferroni correction was applied to adjust for multiple comparisons and distribution of estimation errors across the experimental conditions visually explored using boxplots.

#### Body mass index (BMI)

Participants’ BMI was calculated by dividing weight in kilograms by height squared in metres (kg/m^2^) using self-reported weight and height.

## Results

Characteristics of the 128 participants (51.6% male) are summarised in Table 1. Subjects were predominantly students (71.1%) and born in Australia (60.9%), with a mean age of 29.2 ± 9.3 years and a BMI of 24.0 ± 4.0 kg/m^2^. Foods that were on average consumed at least 2-4 times a week were vegetables M = 1.6, SD =0.8), milk (M = 1.7, SD = 1.3), bread (M = 1.9, SD = 1.1) and cheese (M = 2, SD = 1.1). Cake (M = 4.0, SD = 1.1) and nectarines (M = 4.4, SD = 1.1) were consumed less frequently. Participant characteristics and potential confounders (numeracy, cooking frequency, use of measuring aids at home, knowledge of dietary guidelines (AGHE), consumption frequency of experimental foods and hunger) did not significantly differ between the four experimental conditions.

**Table 1:** Participant characteristics

		Total (*N* = 128)		Measuring cup (*N* = 36)		IFU (*N* = 31)		Modelling clay (*N* = 31)		No aid (*N* = 30)	
		*Mean*	*SD*	*Mean*	*SD*	*Mean*	*SD*	*Mean*	*SD*	*Mean*	*SD*
Age [years]		29.2	9.3	28.2	7.4	29.7	11.5	29.1	8.2	29.8	10.4
BMI [kg/m^2^]^a^		24.0	4.0	23.4	3.4	23.4	3.5	23.9	3.9	25.4	3.1
Subjective numeracy score^b^		4.8	0.8	5.0	0.7	4.8	0.8	4.6	0.9	4.7	0.7
Hunger level^c^		3.2	1.3	3.2	1.3	3.3	1.4	2.9	1.2	3.2	1.4
Cooking skills^d^	Cooking from scratch	1.8	1.0	1.9	1.1	1.8	0.8	1.8	1.0	1.8	1.0
	Baking	3.6	0.8	3.8	0.7	3.5	0.9	3.5	1.0	3.7	0.7
	Cooking ready meals	3.4	1.1	3.3	1.1	3.4	1.2	3.4	1.2	3.5	1.0
Use of measure-ment aids^d^	Measuring cup	2.7	1.1	2.8	1.2	2.6	1.1	2.6	1.1	2.6	1.2
	Scale	3.8	1.3	3.7	1.4	4.0	1.4	3.7	1.3	3.7	1.3
	Other aids	3.6	1.6	3.8	1.6	3.6	1.6	3.0	1.7	4.0	1.5
		*N*	%	*N*	%	*N*	%	*N*	%	*N*	%
Gender	Female	62	48.4	17	47.2	15	48.4	15	48.4	15	50.0
	Male	66	51.6	19	52.8	16	51.6	16	51.6	15	50.0
Country of birth	Australia	78	60.9	22	61.1	16	51.6	18	58.1	22	73.3
	Other countries	50	39.1	14	38.9	15	48.4	13	41.9	8	26.7
Student	Yes^e^	91	71.1	25	69.4	25	80.6	23	74.2	18	60.0
	No	37	28.9	11	30.6	6	19.4	8	25.8	12	40.0

^a^Body Mass Index (BMI) = Weight / Height^2^ [kg/m^2^]. Weight and height were self-reported by participants

^b^The subjective numeracy score is the average score of the eight questions of the Subjective Numeracy Scale (Fagerlin et al., [26])

^c^Hunger level was measured on a six-point scale (1 = Not hungry at all; 6 = Very hungry)

^d^Cooking skills and use of measurement aids were measured on a five-point scale (1 = Daily; 2 = Several times per week; 3 = Several times per month; 4 = Once a month or less; 5 = Never)

^e^Includes full-time and part-time students

### Comparison of errors depending on aid

Relative estimation errors significantly differed between study groups (*H*(3) = 28.48, *P* < .01) (Table 2). The smallest estimation error was in the group using the IFU™ (*Mdn* = 18.9%, *IQR* = 50.2%) and largest for the measuring cup (*Mdn* = 87.7%, *IQR* = 56.1%). The median error for the group without a portion size estimation aid (PSEA) was 23.5% (*IQR* = 79.8%) and 44.8% (*IQR* = 41.9%) for the modelling clay. Estimation errors were significantly larger with the measuring cup compared to the IFU™ (*U* = 183.00, *P* < .01), the modelling clay (*U* = 278.00, *P* < .01) and the group with no estimation aid (*U* = 258.00, *P* < .01). The later three experimental conditions did not have significantly different estimation errors (*P* > .05).

**Table 2:** Relative estimation error by food (*N* = 17) and experimental condition

Food (portion size)	Total (*N* = 128)		Measuring cup (*N* = 36)		IFU™ (*N* = 31)		Modelling clay (*N* = 31)		No aid (*N* = 30)		F-Test
	*Mdn*	*IQR*	*Mdn*	*IQR*	*Mdn*	*IQR*	*Mdn*	*IQR*	*Mdn*	*IQR*	*H(3)*
Bread	−2.6	71.8	12.2	92.1	−17.8	61.6	−1.4	41.1	−24.9	87.1	3.25
Pasta	80.4	142.9	132.3^a^	101.0	55.0^b,c^	103.4	106.7^a,b^	118.9	4.9^c^	100.1	33.22**
Rice (medium)	17.6	81.9	38.4	57.7	−11.5	59.0	6.3	73.8	25.5	120.8	11.47
Mixed vegetables	74.2	93.6	119.3^a^	109.6	49.8^b^	74.9	68.5^b^	112.3	72.9^b^	152.3	24.01**
Potatoes	32.9	88.1	81.3^a^	93.3	32.5^b,c^	66.3	45.8^a,b^	66.3	−11.1^c^	67.8	32.30**
Lettuce	152.5	297.4	294.7^a^	302.6	168.8^b^	201.6	168.8^b^	201.6	−55.6^c^	44.4	68.96**
Strawberry	39.9	104.9	95.9^a^	135.1	32.9^b,c^	87.4	74.8^a,b^	69.9	27.3^c^	97.7	16.82*
Nectarine	−10.7	58.7	38.1^a^	52.9	−29.3^b^	21.2	−5.7^c^	47.1	−28.8^b,c^	57.4	34.84**
Apple	−12.5	48.6	11.6^a^	33.5	−37.1^b^	28.6	−24.0^b,c^	35.7	2.0^a,c^	71.4	33.39**
Steak	53.5	90.3	113.2^a^	146.6	25.6^b^	54.6	50.2^a,b^	65.5	23.4^b^	84.7	17.09*
Chicken (medium)	73.3	122.9	116.6^a^	86.7	33.2^b^	72.1	33.2^b^	44.4	107.5^a^	176.6	29.43**
Nuts & dried fruit	4.9	69.8	11.5	74.3	−4.7	71.5	−4.7	42.9	50.0	103.8	8.85
Milk	5.5	32.2	17.2^a^	23.4	−9.9^b^	18.8	2.1^a^	36.0	13.8^a^	45.5	24.50**
Grated cheese	−7.9	58.8	20.2	31.3	−26.3	46.1	−26.3	43.0	0.7	73.8	10.75
Cake	29.8	69.7	35.1^a^	42.2	−13.5^b^	47.6	29.8^a^	62.7	21.2^a,b^	148.5	21.56**
Chocolate	33.8	108.7	101.3^a^	129.9	3.7^b,c^	66.9	67.2^a,b^	133.8	−8.5^c^	76.2	22.17**
French fries (medium)	79.9	109.1	129.3^a^	89.9	61.0^b^	92.0	84.0^a,b^	115.0	48.8^b^	120.0	20.08**
All foods	44.1	69.7	87.7^a^	56.1	18.9^b^	50.2	44.8^b^	41.9	23.5^b^	79.8	28.48**

*Note*: Differences between study groups were investigated using the Kruskal-Wallis test with the Bonferroni correction for 17 comparisons (* *P* < .05, ** *P* < .01). Post hoc comparisons were performed using the Mann-Whitney test with the Bonferroni correction for six comparisons. Different superscript letters indicate significant differences between groups

### Comparison of errors depending on food

All foods were on average overestimated with the measuring cup, whereas there was both over- and underestimation for the IFU™, the modelling clay and the group with no PSEA. Overestimation in all four study groups was found for six foods (pasta, mixed vegetables, chicken, French fries, strawberry and steak). The remaining 11 foods (bread, rice, potatoes, lettuce, nectarine, apple, nuts & dried fruit, milk, cheese, cake and chocolate) were both over- and underestimated depending on the aid used. Relative errors were compared between the four experimental conditions for each food (Table 2). For four foods there was no significance between conditions (bread, rice, mixed nuts & dried fruit and grated cheese), with a median error across all four conditions ranging from −7.9% (grated cheese) to +17.6% (rice). The estimation errors for the remaining 13 foods differed significantly between the study groups. Post hoc comparisons revealed that estimation errors for the IFU™ and the measuring cup significantly differed for all 13 foods. Compared to the measuring cup, twelve foods were estimated more accurately using the IFU™, with only one food assessed less accurately (apple). The modelling clay performed better than the IFU™ for the nectarine and milk. The group with no PSEA accurately estimated the apple, pasta and grated cheese and poorly estimated mixed vegetables, lettuce, chicken and French fries. Individual estimation errors varied greatly for all estimation aids and foods.

**Fig. 2:**
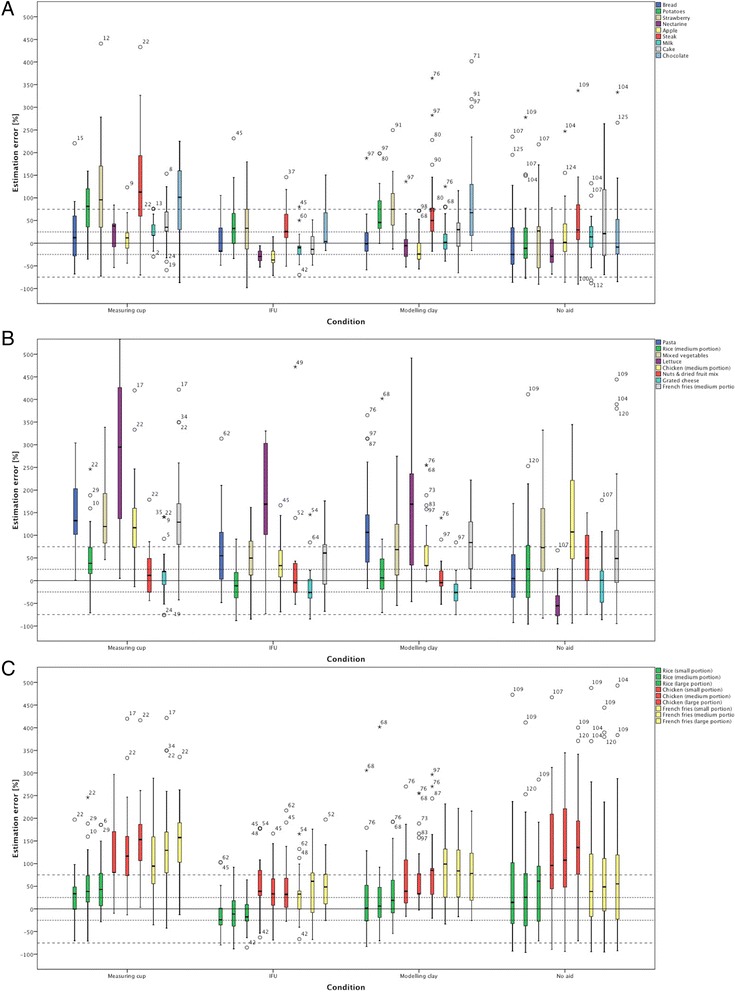
Variation of the relative estimation error by experimental condition **a**) for solid and liquid foods, **b**) for amorphous foods and **c**) for different portion sizes

Estimates made with the IFU™ generally had lower variation compared to the other three study groups, with solid and liquid foods (Fig. 2a) being more accurately estimated than amorphous foods (Fig. 2b). Lettuce was poorly estimated in all four experimental conditions, with large overestimation for the measuring cup, the IFU™ as well as the modelling clay (median estimation error of 294.7%, 168.8% and 168.8% respectively) and considerable underestimation for the group with no PSEA (−55.6%). When estimates for lettuce are excluded, median estimation errors for the group using the IFU™ ranged from −37.1% (apple) to +61.0% (French fries). In contrast, errors for the measuring cup ranged from +11.5% (mixed nuts & dried fruit) to +132.3% (pasta), for the modelling clay from −26.3% (grated cheese) to +106.7% (pasta) and for the group with no estimation aid from −28.8% (nectarine) to +107.5% (chicken).

Estimation accuracy was also assessed by classifying the absolute errors into estimates within 25% and 75% of the objective food amount. The proportion of total estimations within this range was greatest for the group using the IFU™ (38.8%; 81.1%) in comparison to groups using the modelling clay (31.6%; 70.5%), the measuring cup (19.6%; 51.1%) and no PSEA (24.3%; 65.5%).

### Comparison of errors for different portion sizes

The influence of portion size on estimation accuracy was investigated for rice, chicken and French fries (Fig. 2c and Table 3). For each of these foods, participants estimated three different portion sizes (small, medium, large). The estimation errors tended to increase with increasing portion size. However, significant differences between portion sizes were only observed for chicken and French fries in the group using the measuring cup (*P* < .05). The large portion of chicken had a significantly larger estimation error compared to the small portion (*T* = 128, *P* < .01). The medium and large portions of French fries had significantly larger estimation errors than the small portion of French fries (*T* = 158, < .05; *T* = 137, *P* < .01).

**Table 3:** Relative estimation error by experimental condition, food (*N* = 3) and portion size (small, medium, large)

Condition	Food	Small portion		Medium portion		Large portion		F-test
		*Mdn*	*IQR*	*Mdn*	*IQR*	*Mdn*	*IQR*	χ^2^ (2)
Measuring cup (*N* = 36)	Rice	33.7	49.5	38.4	57.7	42.8	71.4	3.72
	Chicken	80.5^a^	90.3	116.6^a,b^	86.7	153.1^b^	81.4	14.39*
	French fries	94.3^a^	103.6	129.3^b^	89.9	157.6^b^	87.1	11.17*
IFU™ (*N* = 31)	Rice	−23.9	38.0	−11.5	59.0	−17.8	36.5	0.84
	Chicken	38.8	55.5	33.2	72.1	32.2	76.7	3.94
	French fries	32.7	46.4	61.0	92.0	48.6	74.3	3.94
Modelling clay (*N* = 31)	Rice	1.4	81.1	6.3	73.8	18.7	80.4	5.10
	Chicken	38.8	120.3	33.2	44.4	85.1	60.8	3.68
	French fries	99.1	112.8	84.0	115.0	78.3	104.0	3.16
No aid (*N* = 30)	Rice	14.6	138.1	25.5	120.8	61.4	121.4	0.47
	Chicken	95.9	170.1	107.5	176.6	135.4	117.7	0.47
	French fries	38.3	145.2	48.8	120.0	55.0	145.2	1.67

*Note*: Differences between several related groups were investigated using Friedman’s ANOVA with the Bonferroni correction for 12 comparisons (* *P* < .05). Post hoc comparisons were performed using the Wilcoxon signed-rank test with the Bonferroni correction for three comparisons. Different superscript letters indicate significant differences between groups

### Usability of estimation aids

Participants evaluated helpfulness and ease-of-use for the three estimation aids (Additional file 1: Table S3). The reported helpfulness of the PSEAs was comparable (*H*(2) = 5.25, *P* > .05) between groups. Significant differences were found for ease-of-use. The modelling clay was reported to be significantly easier to use compared to the IFU™ (*U* = 214.00, *P* < .01) and the measuring cup (*U* = 202.00, *P* < .01). In the questionnaire, participants could also comment on usability of the aid in an open-ended question. In general, participants found it difficult to estimate foods of shapes and sizes, which differed from the provided aid, for example non-cube shaped foods using the IFU™. They also had difficulties estimating the volume of irregular shaped foods such as French fries or mixed vegetables. Measuring cups were considered to be useful for liquids such as milk and compact foods such as rice. Participants using the IFU™ appreciated that the cube could be subdivided into smaller units. However, some people stated that they would need training to become more familiar with using it. The modelling clay was seen as helpful and easy because it could be manipulated to replicate food shape and size.

Additional file 1: Table S4 summarizes the foods, which participants considered the easiest to estimate. Foods they found difficult to estimate were similar across the four study groups. They were mostly amorphous, for example French fries, mixed vegetables and rice. The strawberry was perceived to be difficult to estimate using the measuring cup. Foods perceived the easiest to estimate differed between the four experimental conditions. Participants found rice and milk the easiest to estimate using a measuring cup; chocolate using the IFU™ or the modelling clay; and steak, milk and chicken breast by estimating the weight with no PSEA.

## Discussion

The IFU™ had the lowest mean estimation error and error variation compared to the measuring cup, the modelling clay and the group that estimated weight with no PSEA. However, none of the study subjects actual disassembled the IFU cube. Future studies should investigate whether training with the IFU could further enhance estimation accuracy. In the future, a standardised food volume unit such as the IFU™ may serve as a 3D reference object on food photographs or potentially within smartphone applications.

Modelling clay has been previously used for portion size estimation [27]. It provided the most accurate estimates in two studies included in that paper (absolute error of 33.2% and 40.6%) compared to measuring cups and spoons, as well as household objects such as tennis balls or decks of cards. The absolute error for modelling clay in the present study was slightly higher (*Mdn* absolute error 54%). The measuring cup performed worst in the current study. All foods were on average overestimated using the measuring cup, with the median relative error ranging from 11.5% (mixed nuts & dried fruit) to 274.7% (lettuce). This is in agreement with other studies that had concluded that measuring cups performed worst compared to several other PSEAs tested [27, 28]. Bernal-Orozco et al. also found an overestimation of portion size using measuring cups [28]. In their study, all food groups were on average overestimated, with errors also being lowest for nuts/oilseeds and highest for meats. This indicates that measuring cups are a useful tool to estimate nut portion size. Meat products might be poorly estimated with cups due to their irregular shape [29]. Lettuce might be difficult to estimate, as the volume of salad leaves is very small. Foods with small volumes, such as spreads were found to be overestimated in other studies as well [22, 30–32].

A source of estimation errors in the measuring cup condition could be subjects’ estimation strategy. Some of them reported imagining how much food they could fill in the cup with estimates including air or space in foods that cannot be compressed easily, unless mashed or chopped up (e.g. mixed vegetables or chicken). In contrast, subjects using the IFU™ or the modelling clay predominantly reported accounting for air in between single food items and only compared the actual food volume with the volume of the estimation aid. This might explain the overestimation with measuring cups, indicating that people need specific instructions on how to estimate food portion sizes (i.e. loose vs. compressed/without air). Furthermore, we provided only one measuring cup, rather than a set of measuring cups, which may have been helpful in estimating different volumes. However, the cup provided had measurement increments on it and the IFU cube had visible subunits. Also, we note that none of the participants disassembled the cube in the current study. Future studies need to test, whether training participants on how to use the cube for measuring and estimating volume can further enhance volume estimation accuracy.

### Food weight estimation (no aid)

The group without an estimation aid had comparable estimation errors to people using the IFU™. The small estimation error might be due to the fact that participants did not estimate food volume, but weight. Weight estimation might be easier for consumers as most products in supermarkets contain food labels with information on product weight, whereas volume information is usually restricted to liquids. In line with this, subjects mentioned that their experience in cooking or grocery shopping helped them estimate food weight.

### Portion size and food characteristics

In the present study estimation errors tended to increase with increasing portion size. However, significant differences of errors between portion sizes were only found for chicken and French fries, in the group using the measuring cup. Previous studies have reported an influence of portion size on estimation accuracy. Small portions tended to be overestimated and large portions underestimated, a phenomenon called ‘flat-slope’, or the tendency to avoid extreme response categories [20–22, 33]. Another common finding is that large portions are less accurately estimated than small portions [34, 35], potentially because it is easier to estimate food portions similar in size to the PSEA. However, the two studies that identified this effect were conducted using food photographs [34, 35]. Photographs usually depict a whole range of portion sizes consumed so potentially there may not have been a large deviation from the actual food portion to assess. Further, amorphous foods were reported to be associated with higher estimation errors compared with solid and liquid foods [22, 36]. This is in agreement with the findings of the present study.

### Usability of estimation aids

The foods participants perceived the easiest and most difficult to estimate were those foods with the lowest and highest estimation errors respectively. This indicates that people’s perception generally corresponds to actual estimation errors. Participants mentioned that they found measuring cups the most useful for compact foods and liquids, while those in the IFU™ and modelling clay conditions reported that chocolate was the easiest to estimate, indicating that cubic or rectangular-shaped objects were easier to estimate with cubic PSEAs than other forms. People estimating food weight (the condition with no PSEA) found steak, milk and chicken breast the easiest to assess. Those foods are usually bought by weight (e.g. meat) or in defined amounts (e.g. 1 L of milk), which probably enhances weight estimation. Amorphous foods such as French fries or mixed vegetables were considered the most difficult to estimate across all four experimental conditions. This is in agreement with previous studies reporting that amorphous foods often have higher estimation errors compared to solid and liquid foods [22, 36].

The use of internationally standardised measurement units, consistent dietary recommendations and unambiguous terminologies could help to avoid consumer confusion, enhance people’s ability to accurately estimate portion sizes and improve dietary intakes [11]. Practical tools, clear indications about the aim of PSEAs (optimising health vs. aiding weight loss) as well as detailed instructions on how to use them are recommended [37]. Despite the use of PSEAs, estimation errors with the IFU were still large for some foods. Participants in the current study received minimal instructions only on how to use the aids, with no specific training. Previous research indicates that portion size education/training using PSEAs [36] improves estimation accuracy.

### Study limitations

The current study has several limitations. Firstly, only single foods were included, except for the mixed vegetables consisting of broccoli, cauliflower, carrots and beans. However, most dishes such as curries or stir-fries have various components. Estimation errors are expected to be higher for composite dishes than single foods. So far, there are no validated alternative PSEAs for mixed dishes [37]. Further, future studies should also include foods served in bowls such as porridge or soup. Secondly, the sample was relatively young (mean age of 29.2 ± 9.3 years), well educated (71.1% university students) and from one geographical location (Newcastle, Australia). The influence of age and education level has been assessed in several studies with most concluding that there is no effect of age [20, 22, 25, 32, 34, 38] or education level [20, 22, 34]. The influence of ethnicity on estimation accuracy has only been evaluated in one study in children [25] with no significant effect found. Thirdly, there was no group who estimated volume without the use of a PSEA in the current study, as food weight was estimated. However, previous research indicates that people’s ability to estimate volume without a reference object is limited and visual tools generally enhance accuracy in estimating food portion sizes [5].

## Conclusion

The current study provides evidence that the IFU™, a new measurement cube with standardised dimensions of 64 cm^3^, can be a useful tool for food volume estimation. The IFU™ performed best of the PSEAs tested, with the lowest variation in estimation errors. However, consumers may require instructions and/or training to become familiar with the IFU™. Overall, the IFU™ was perceived as a helpful tool to estimate food volumes. Further studies should investigate whether training including the IFU™ enhances estimation accuracy and can assist with food volume estimation in everyday situations. It would also be relevant to investigate the performance of the IFU™ with composite dishes, including curries or stir-fries and with people from other cultural backgrounds and differing eating habits.

## Additional file

Additional file 1: Figure S1. Food portions (s; solid, a; amorphous; l; liquid). **Table S1.** Details of food portions. **Table S2.** Portion size estimation aids used in the experiment. **Table S3.** Helpfulness and ease of use of the three PSEAs used in this study**. Table S4.** Foods subjects found the easiest/most difficult to estimate by experimental condition. (DOCX 761 kb)
