# Supplementary material for: The international food unit: a new measurement aid that can improve portion size estimation
Source: Int J Behav Nutr Phys Act. 2017 Sep 12;14:124. doi: 10.1186/s12966-017-0583-y (PMC5596841; doi:10.1186/s12966-017-0583-y)
Supplement: Additional file 1: Figure S1. — Food portions (s; solid, a; amorphous; l; liquid). Table S1. Details of food portions. Table S2. Portion size estimation aids used in the experiment. Table S3. Helpfulness and ease of use of the three PSEAs used in this study. Table S4. Foods subjects found the easiest/most difficult to estimate by experimental condition. (DOCX 761 kb) [file 12966_2017_583_MOESM1_ESM.docx]

Additional file Tables and Figures

Additional file: Figure S1: Food portions (s; solid, a; amorphous; l; liquid).

(1) bread, s; (2) pasta, a; (3)-(5) rice, a; (6) mixed vegetables, a; (7) boiled potatoes, s; (8) lettuce, s; (9) strawberry, s; (10) nectarine, s; (11) apple, s; (12) steak, s; (13)-(15) chicken, a; (16) mixed nuts & dried fruit, a; (17) milk, l; (18) grated cheese, a; (19) cake, s; (20) milk chocolate, s; and (21)-(23) French fries, a.


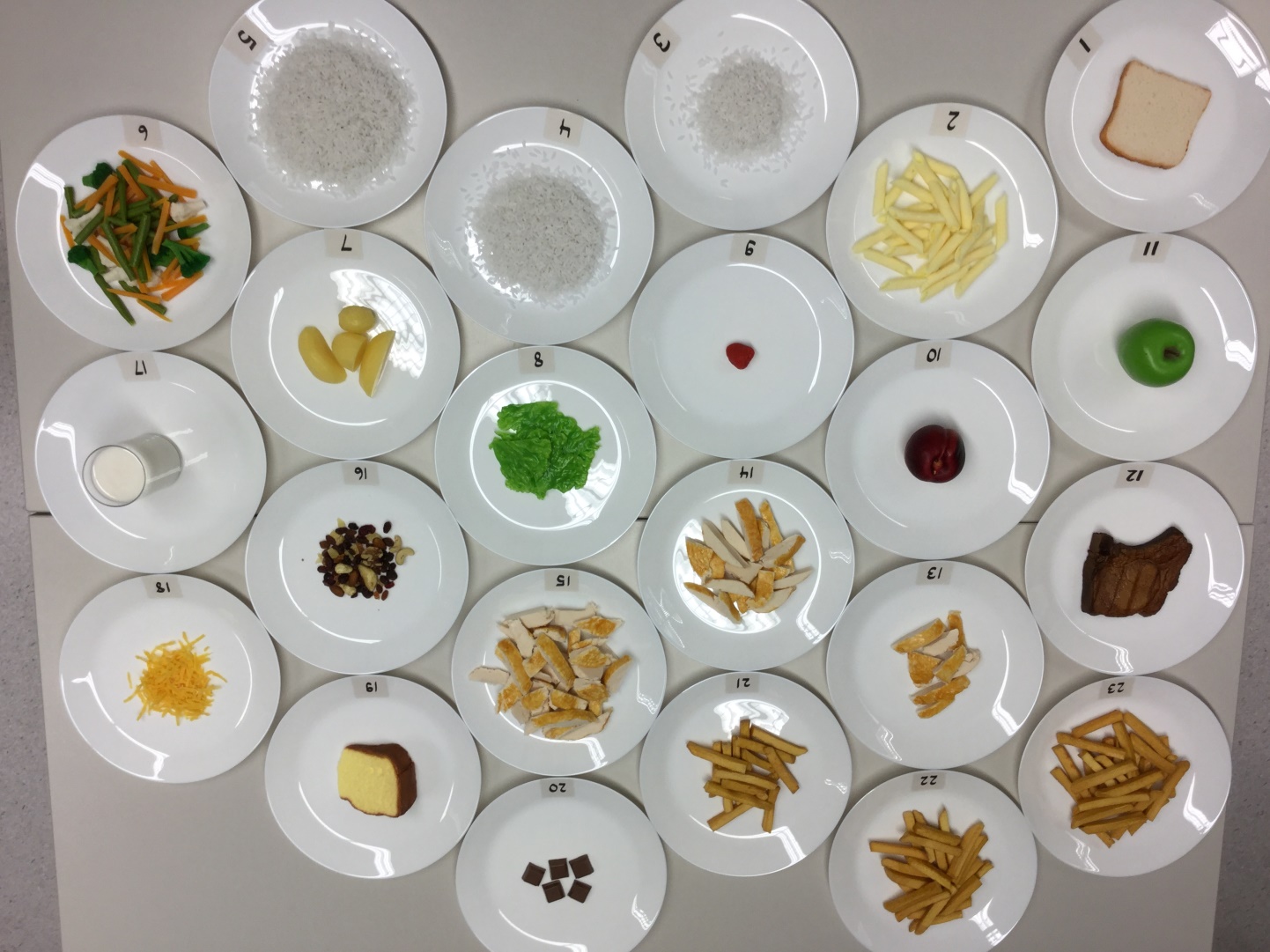


Additional file : **Table S1:** Details of food portions.

| Food | Portion size | Weight of fake food [g] | kJ per fake food item | Estimated weight corresponding to real food [g] | Energy [kJ] ^g^ | Food group ^h^ | Volume [mL] ^i^ | Proportion of a cup (250 mL) | Proportion of the IFU (64 mL) |
| --- | --- | --- | --- | --- | --- | --- | --- | --- | --- |
| Bread (white) | 1 slice | 85.0 | 371 | 37.3 | 371 | 1 | 77.9 | 0.31 | 1.22 |
| Pasta | Typical ^b^ | 124.5 | 455 ^d,e^ | 111.1 | 566 | 1 | 123.8 | 0.50 | 1.93 |
| Rice (white) | Small ^b^ | 78.0 | 667 ^d,e^ | 74.2 | 520 | 1 | 126.2 | 0.50 | 1.97 |
|  | Medium ^b^ | 134.0 | 667 ^d,e^ | 127.5 | 894 | 1 | 216.8 | 0.87 | 3.39 |
|  | Large ^b^ | 216.5 | 667 ^d,e^ | 206.0 | 1444 | 1 | 350.3 | 1.40 | 5.47 |
| Mixed vegetables ^a^ | Typical ^b^ | 186.0 | 99.5 ^d,e^ | 115.7 | 185 | 2 | 170.9 | 0.68 | 2.67 |
| Potatoes | Typical ^b^ | 109.0 | 289 ^d,e^ | 112.5 | 315 | 2 | 96.6 | 0.39 | 1.51 |
| Lettuce | 6 leaves | 26.0 | 6 ^e^ | 45.0 | 36 | 2 | 23.8 | 0.10 | 0.37 |
| Strawberry | 1 piece | 9.5 | 12 | 11.0 | 12 | 3 | 9.2 | 0.04 | 0.14 |
| Nectarine | 1 piece | 162.0 | 284 | 156.7 | 284 | 3 | 135.7 | 0.54 | 2.12 |
| Apple | 1 piece | 266.5 | 297 ^e^ | 147.0 | 297 | 3 | 223.9 | 0.90 | 3.50 |
| Steak (beef) | 1 piece | 131.0 | 1216 ^e^ | 162.3 | 1216 | 4 | 117.2 | 0.47 | 1.83 |
| Chicken breast | Small ^b^ | 76.0 | 636 ^d,e^ | 48.5 | 483 | 4 | 69.2 | 0.28 | 1.08 |
|  | Medium ^b^ | 158.5 | 636 ^d,e^ | 101.2 | 1008 | 4 | 144.2 | 0.58 | 2.25 |
|  | Large ^b^ | 266.0 | 636 ^d,e^ | 169.9 | 1692 | 4 | 242.0 | 0.97 | 3.78 |
| Mixed nuts & dried fruit | Snack size | - | - | 40.0 | 738 | 4 | 67.1 | 0.27 | 1.05 |
| Milk (cow) | 1 glass | - | - | 219.6 ^f^ | 617 | 5 | 213.2 | 0.85 | 3.33 |
| Cheddar cheese (grated) | A hand full | 15.5 | 3094 ^d^ | 28.8 | 479 | 5 | 52.1 | 0.21 | 0.81 |
| Cake (uniced) | 1 slice | 130.5 | 1227 | 82.5 | 1227 | D | 148.0 | 0.59 | 2.31 |
| Milk chocolate | Typical ^b^ | 21.0 | 3447 ^d^ | 32.8 | 724 | D | 19.1 | 0.08 | 0.30 |
| French fries | Small ^c^ | 107.5 | 660 ^d,e^ | 72.3 | 710 | D | 96.5 | 0.39 | 1.51 |
|  | Medium ^c^ | 155.0 | 660 ^d,e^ | 104.2 | 1023 | D | 139.1 | 0.56 | 2.17 |
|  | Large ^c^ | 192.0 | 660 ^d,e^ | 129.0 | 1267 | D | 172.3 | 0.69 | 2.69 |

*Note*: The nuts & dried fruit mix was real food (Woolworths Limited, Australia). All other foods were very authentic food replicas (Döring GmbH, Germany).

^a^ Mixed vegetables contained broccoli, cauliflower, carrots, and beans.

^b^ Typical, small, medium, and large portions sizes were determined according to Australian adults’ perception of the corresponding or similar foods (Collins et al., 2015).

^c^ Portions sizes of French fries were taken from an international chain of fast food restaurants (McDonalds Australia, 2016).

^d^ For continuous food items, the theoretical energy value of 100 g fake food product is given.

^e^ Conversion factors have been established (Bucher et al., 2013). Fake foods without a conversion factor were compared to their corresponding real food products (Coles Supermarkets Australia Pty Ltd., Australia).

^f^ Weight of real milk was calculated by multiplying its volume with its density of 1.03 g/mL (Charrondiere et al., 2012).

^g^ Calculations of the energy content were based on the Australian Food, Supplement, and Nutrient Database (AUSNUT) 2011-13 (Food Standards Australia New Zealand

(FSANZ), 2016).

^h^ Food groups are based on the Australian Guide to Healthy Eating (AGHE): (1) breads and cereals; (2) vegetables; (3) fruits; (4) meat, poultry and legumes; (5) dairy; and (D)

discretionary foods (National Health and Medical Research Council (NHMRC), 2016).

^i^ Volume was established by water replacement except for rice, mixed nuts and dried fruit, grated cheese (without water) and milk (estimation using reference line at 2 dL on

the glass).

Additional file : Table S2: Portion size estimation aids used in the experiment.

| Estimation aid | Description | Picture |
| --- | --- | --- |
| International Food Unit^TM^ (IFU^TM^) | This 64 cm^3^ cube can be subdi­vided into eight smaller cubes of 8 cm^3^ in order to estimate smaller food items. The cube is made of Acrylonitrile Butadiene Styrene (ABS) and was 3-D printed using Fused Deposition Modelling (FDM). | 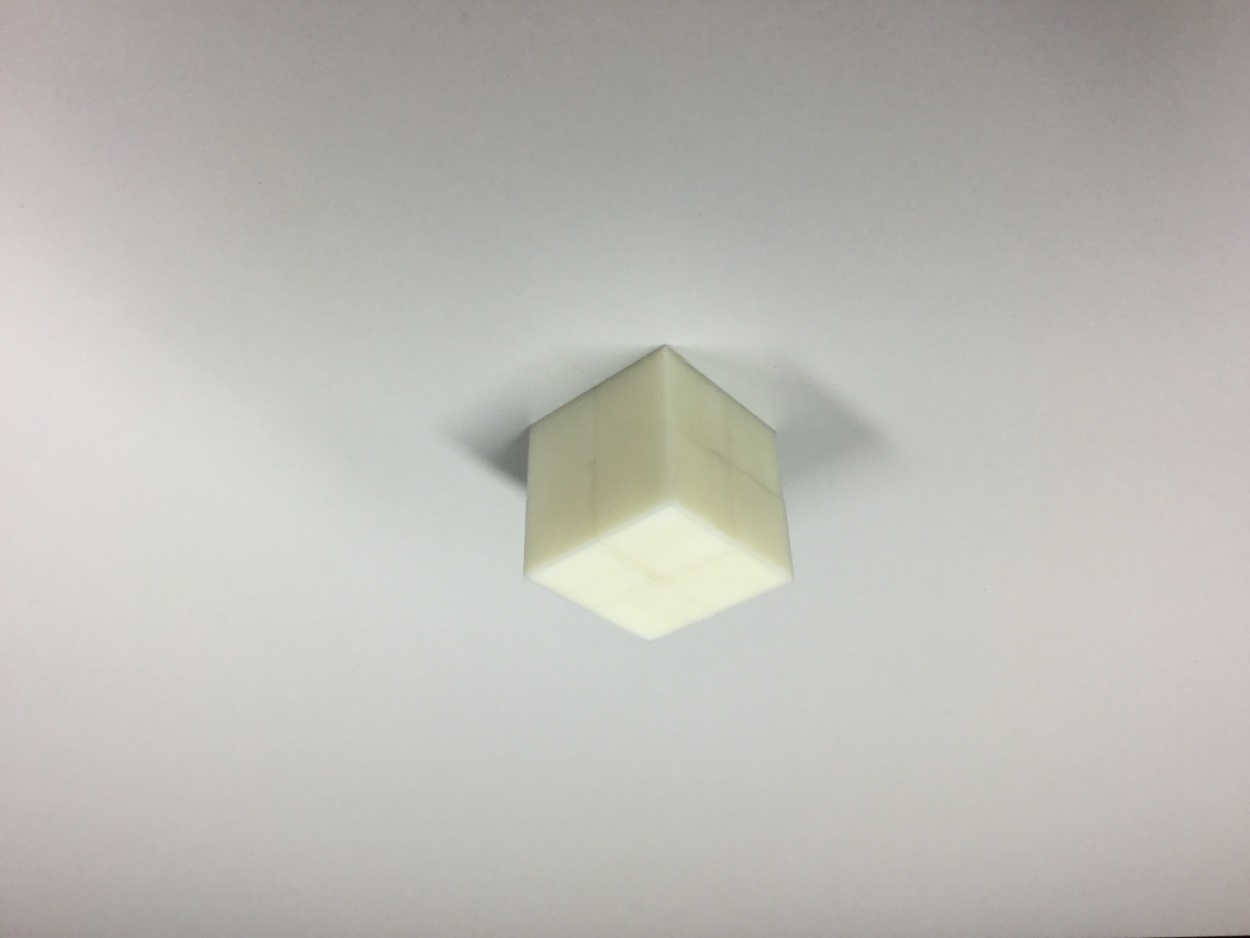 |
| Modelling clay | This cube was made of modelling clay and can be moulded and reshaped. It had the same vol­ume as the IFU^TM^ (64 cm^3^). | 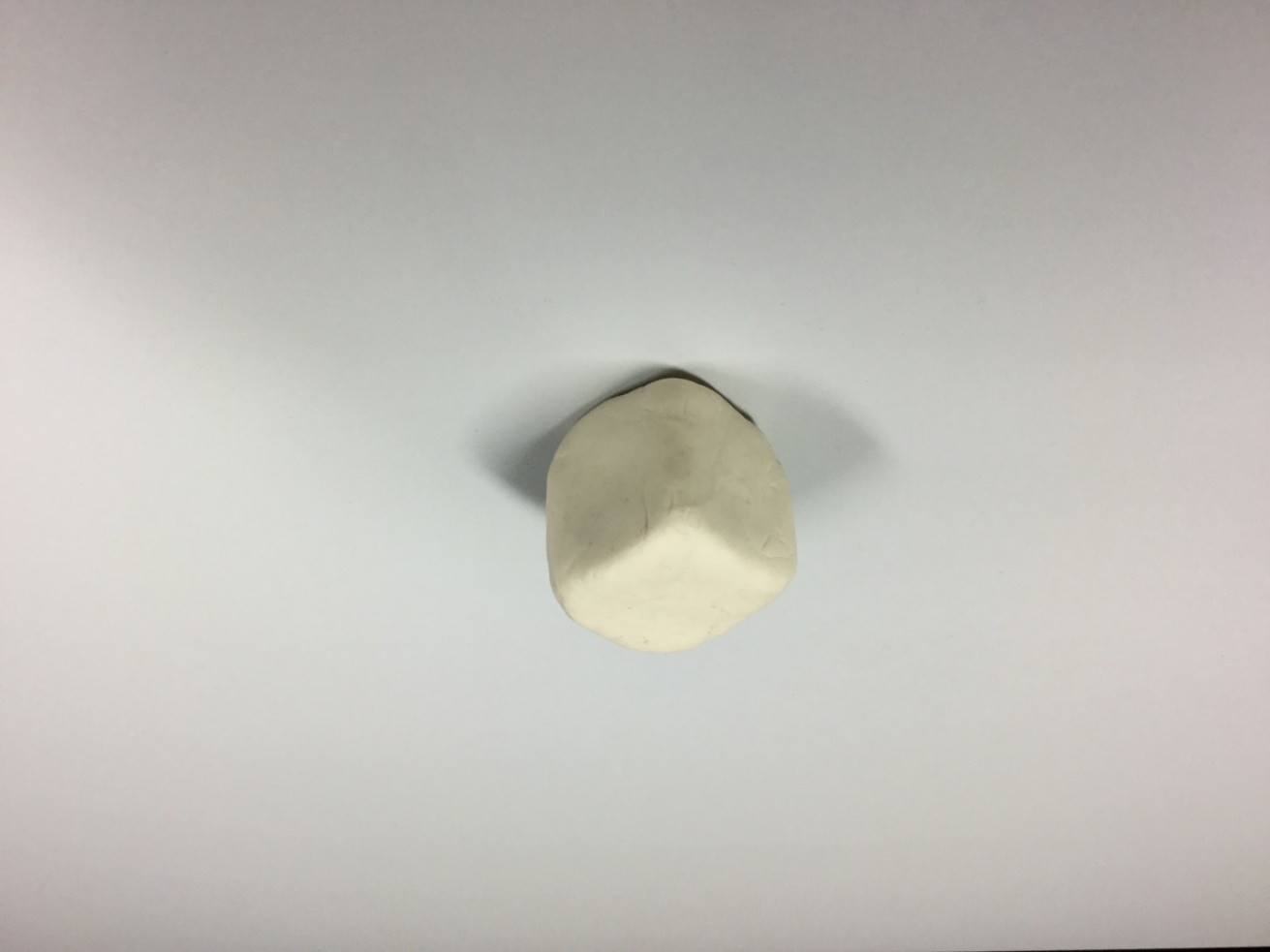 |
| Measuring cup (Australian) | We used the standard measuring cup in Australia, which has a volume of 250 mL. Dash lines at the side of the cup help to measure or estimate smaller amounts of food. | 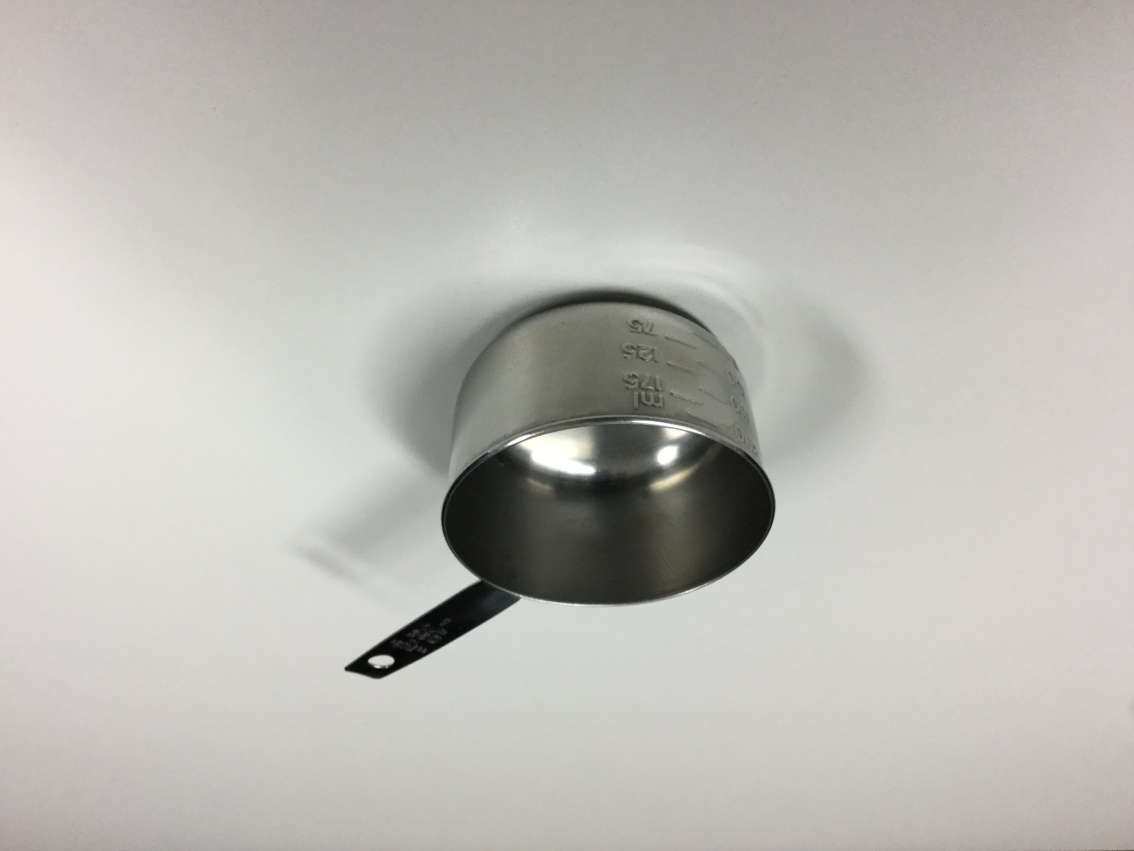 |

Additional file : Table S3: Helpfulness and ease of use of the three PSEAs used in this study.

|  | Measuring cup  (*N* = 33) | | | IFU^TM^  (*N* = 30) | | | Modelling clay  (*N* = 27) | | | F-Test |
| --- | --- | --- | --- | --- | --- | --- | --- | --- | --- | --- |
|  | *Mdn* | *Mean* | *SD* | *Mdn* | *Mean* | *SD* | *Mdn* | *Mean* | *SD* | *H(2)* |
| The aid helped me to estimate portion size. | 4 | 3.79 | 0.89 | 4 | 3.73 | 0.87 | 4 | 4.22 | 0.70 | 5.25 |
| The aid was easy to use to estimate portion size. | 3 | 2.97 ^a^ | 0.88 | 3 | 3.07 ^a^ | 0.98 | 4 | **3.93** ^b^ | 0.87 | 16.34** |

*Note*: Statements were measured on a five-point scale (1 = strongly disagree; 5 = strongly agree). Differences between groups were investigated using the Kruskal-Wallis test with the Bonferroni correction for three comparisons (* P < .05, ** P < .01). Post hoc comparisons were performed using the Mann-Whitney test with the Bonferroni correction for three comparisons. Different superscript letters indicate significant differences between groups.

Additional file : Table S4: Foods subjects found the easiest/most difficult to estimate by experimental condition.

| Condition | Foods the easiest to estimate  (% within condition) | Foods the most difficult to estimate (% within condition) |
| --- | --- | --- |
| Measuring cup (*N* = 36) | Rice (69.4%)  Milk (25.0%) | French fries (38.9%)  Strawberry (22.2%) |
| IFU^TM^ (*N* = 31) | Chocolate (29.0%) | French fries (22.6%)  Mixed vegetables (22.6%) |
| Modelling clay (*N* = 31) | Chocolate (22.6%) | Mixed vegetables (35.5%)  Rice (23.1%) |
| No aid (*N* = 30) | Steak and milk (23.3% each)  Chicken breast (20.0%) | Rice (26.7%)  French fries (20.0%) |
